# Time to Wake Up: Epigenetic and Small-RNA-Mediated Regulation during Seed Germination

**DOI:** 10.3390/plants10020236

**Published:** 2021-01-26

**Authors:** Eduardo Luján-Soto, Tzvetanka D. Dinkova

**Affiliations:** Departamento de Bioquímica, Facultad de Química, Universidad Nacional Autónoma de México, Ciudad de México 04510, Mexico; eduardolujan@quimica.unam.mx

**Keywords:** DNA methylation, dormancy, germination, histone modification, small RNAs

## Abstract

Plants make decisions throughout their lifetime based on complex networks. Phase transitions during seed growth are not an exception. From embryo development through seedling growth, several molecular pathways control genome stability, environmental signal transduction and the transcriptional landscape. Particularly, epigenetic modifications and small non-coding RNAs (sRNAs) have been extensively studied as significant handlers of these processes in plants. Here, we review key epigenetic (histone modifications and methylation patterns) and sRNA-mediated regulatory networks involved in the progression from seed maturation to germination, their relationship with seed traits and crosstalk with environmental inputs.

## 1. Introduction

Seed is a fundamental entity in the life cycle of higher plants that functions to protect the embryo. It senses environmental cues to couple germination with optimal developmental conditions for the new plant. Timing of seed germination is a central trait controlled by complex network of biochemical and molecular mechanisms. The balance between inactive and germinating states impacts not only the offspring viability but also agronomical and industrial features like crop production and yield, harvesting period and post-harvesting processing. Molecular dissection of seed development phase transitions has revealed cell-signaling pathways, hormonal balance interactions, biotic or abiotic stress effects and biomechanical aspects leading to germination arrest and progression. Lately, attention has been directed to learning from large-scale genome reprogramming, where coordinated expression profiles are required for phase transitions during seed life cycle and are often associated with major changes in chromatin structure. Dynamic transcriptional control is endorsed by interactions between epigenetic effectors. Several mutants with known defects in seed maturation, dormancy and germination correspond to genes involved in chromatin structure, DNA methylation and sRNA pathways, evidencing the importance of epigenetic regulation and chromatin dynamic maintenance during these developmental stages. Here we discuss recent knowledge on epigenetic and non-coding small RNA-mediated regulation during late embryonic maturation driving to dormancy acquisition and posterior germination, with particular highlights on interactions between effectors of each regulatory path to achieve these transitions.

## 2. Seed Dormancy and Germination

### 2.1. Securing the Future Plant

**Figure 1:**
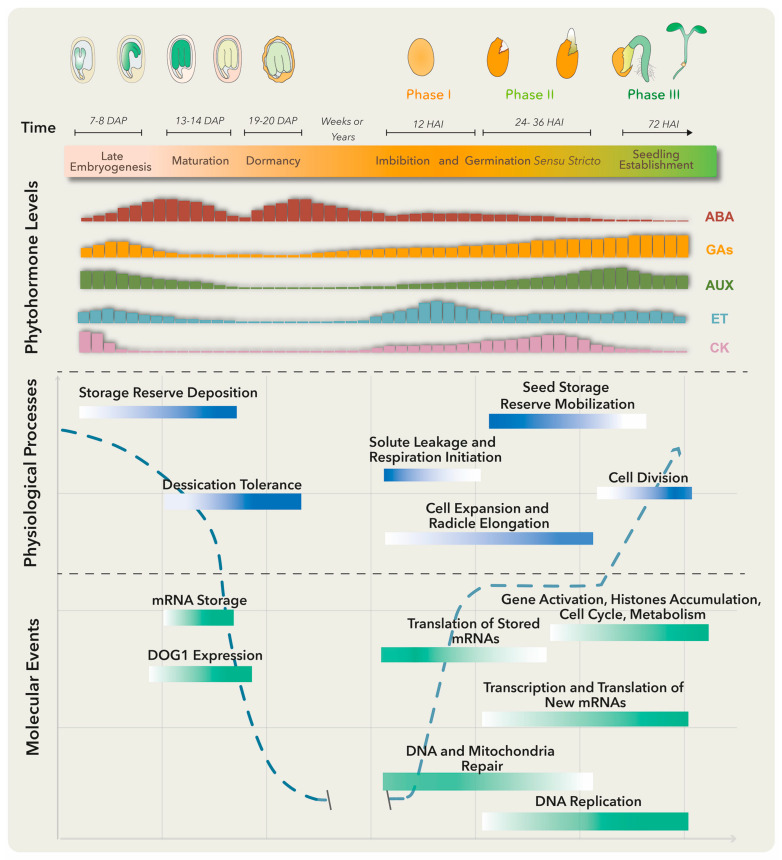
Molecular, biochemical and physiological processes occur from embryo maturation through dormancy to impact on seed germination. The upper panel represents the late seed developmental stages of a dicotiledoneous plant on the left and germination to seedling establishment stages on the right. The lower panels include, from top to bottom, changes in specific phytohormone levels; and physiological processes and molecular events that particularly represent the maturation, dormancy and germination stages. Longer bars refer to higher levels. Abscisic acid (ABA) positively regulates embryo maturation, dormancy induction and maintenance, while gibberellins (GAs) promote release from seed dormancy and germination. Ethylene (ET) production rises after seed soaking with a peak before radicle protrusion. The dashed blue line represents the water level throughout different developmental stages. DAP means days after pollination, and HAI means hours after imbibition.

Several molecular, biochemical and physiological events take place in the embryo and surrounding tissues throughout seed development. These have been grouped into three major stages: embryo development, seed maturation and dormancy (Figure 1). At the late embryogenesis stage, seeds integrate signals and undergo final desiccation to become dormant [1], a state defined as the inability of intact viable seeds to complete germination for a given time period even under optimal environmental conditions [2].

Primary dormancy, referred as dormancy in this review, is established by endogenous factors and influenced by the mother plant’s growing conditions during seed development. In addition, if nondormant or post-dormant seeds face unfavorable conditions for germination, like high temperatures, a secondary dormancy can be activated even after the seeds have been dispersed [3]. Embryo-induced dormancy relates to embryo immaturity or underdevelopment and/or the synthesis of dormancy promotion compounds by this structure [4]. On the other hand, seed-coat dormancy is conducted by tissues surrounding the embryo, such as endosperm, testa and coleorhiza, to restrain embryo growth, leaking of germination inhibitors, water uptake and radicle emergence [4,5]. Numerous treatments could release dormancy depending on the plant species. These include after-ripening (a certain period of storage at room temperature), cold and warm stratifications and seed treatments with smoke, light or nitrate [6]. Breaking seed dormancy promotes the necessary metabolic, hormonal and molecular conditions for germination (Figure 1).

There are several definitions for germination, the most accepted one defining it as the process starting with seed water uptake (imbibition) and concluding with the successful rupture of covering layers by the radicle [7]. Three phases are commonly distinguished during germination: phase I is characterized by rapid water uptake, seed swelling and reshaping, followed by perturbations in membrane structure and leakage of metabolites; phase II comprises a period of slower and stable water uptake concomitant with initial embryo expansion and covering layers weakening; finally, radicle protrusion (germination) marks the end of phase II and the beginning of phase III (a post germinative stage) distinguished by storage product mobilization from the endosperm to the embryo axis, as well as by triggering a second burst of water uptake and seedling growth [4].

### 2.2. Phytohormone Interplay for Seedling Success

Plant hormones are required at specific levels through dormancy, dormant state break and germination. Particularly, abscisic acid (ABA) and gibberellins (GAs) are the master regulators of these processes. ABA positively regulates dormancy induction and maintenance, while GAs promote release from seed dormancy and germination (Figure 1). Other phytohormones such as ethylene, cytokinin (CK), brassinosteroids (BRs), auxins (AUX) and jasmonic acid (JA) have been implicated in certain aspects of seed dormancy and/or germination regulation [6].

Before embryo maturation, a first ABA level increase comes mainly from maternal tissues and plays a key role in embryo growth, while during late maturation, a second peak is observed due to ABA supply from zygotic tissues [8]. When the dormant state is perturbed, the ABA level drops and the germination process starts. Treatments that stimulate dormancy loss can also trigger a substantial ABA reduction by activating genes involved in ABA catabolism [4].

The ABA counterplayers, GAs, comprise a group of compounds that regulate diverse plant processes, including germination and plant growth. The amount of bioactive GAs is reduced at stages where ABA peaks by inactivation reactions, to guarantee normal growth and development (Figure 1). GA excess translates into negative effects, like precocious seed germination and pre-harvesting sprouting or viviparity [9], while GA-deficient mutant seeds from *Arabidopsis* and tomato are unable to germinate unless external GA treatment is applied [10].

Another well-documented hormone involved in seed germination is ethylene (ET). This gaseous hormone plays a key role in seed dormancy release and germination in numerous species [11]. Ethylene antagonizes ABA effects through the regulation of ABA metabolism genes and signaling pathways. Mutants insensitive to ET are hypersensitive to ABA and present extended dormancy periods, while those showing increased ET production exhibit reduced sensitivity to ABA and a decrease in dormancy duration. Furthermore, ET promotes radicle protrusion, germination and seedling establishment by affecting and interacting with GA biosynthesis and signaling pathways [12].

JA and BRs could also impact on seed dormancy and germination. While their role has been poorly explored, evidence suggests that they might affect the ABA/GA balance and action under particular treatments. Briefly, JA exhibits a dual effect on germination depending on plant species. Nondormant *Arabidopsis* seeds display higher JA levels than dormant seeds and the hormone level further decreases upon seed imbibition [13]. On the other hand, cold stratification of wheat seeds triggers the increase in JA endogenous content, which positively regulates the activity of ABA biosynthesis repressors [14]. BRs could also promote seed germination through repressing ABA signaling in *Arabidopsis*. Treatment with BRs was able to rescue the low germination phenotype of both GA-biosynthetic and GA-insensitive mutants [15].

### 2.3. Re-Shaping Quiescent Tissues towards Active Proliferation

To establish a balance between dormancy and germination, a complex regulatory network is built. For dormancy, ABA binds to its receptor proteins and the complex inhibits protein phosphatases 2C (PP2C; negative regulators of dormancy). This allows the activation of SNF1-related protein kinases (SnRK2; positive regulators of dormancy) and downstream target phosphorylation, including transcription factors ABA-insensitive ABI5, ABI3 (B3 family), and ABI4 (Ethylene response factor; ERF family) to activate plant responses [16]. A crosstalk between these and other signaling networks conducts the deposition of storage reserves, acquisition of desiccation tolerance and induction of dormancy. Upon dormancy state breaks in *Arabidopsis thaliana*, different gene expression programs become activated [17]. Genes involved in translation and protein assembly are upregulated [4,17], together with the transcriptional activation of enzymes involved in DNA replication, nitrogen metabolism, mobilization of storage products, cell wall modification, cytoplasmatic membrane-bound vesicle formation and hormone biosynthesis (Figure 1) [5,18]. Furthermore, changes in the seed proteome take place during dormancy release, comprising proteins involved in translation, cellular signaling, energy metabolism and redox status control [19].

During phase I of germination, protein synthesis is supported by pre-existing mRNA even before transcription re-activation. Stored mRNAs that are associated with single ribosomes in the dry seeds and become translationally upregulated in early germination encode proteins involved in redox reactions, glycolysis and translation [20,21]. Other stored mRNAs, accumulated in response to ABA and other environmental factors in late seed maturation and dormancy, are degraded to prevent translation of proteins that function as germination suppressors [21]. Phase I is also associated with DNA and mitochondria repair, required for the success of the germination process [22]. On the other hand, phase II is characterized by the synthesis of new mitochondria, proteins translated from newly transcribed mRNAs and continuous DNA repair [4]. Finally, phase III requires transcriptional activation of genes representing histone families, cell cycle and metabolic pathways to promote DNA synthesis, cell division and radicle elongation (Figure 1) [23]. Such gene expression program switches are finely regulated by the chromatin architecture and epigenetic modifications.

## 3. Main Epigenetic Modifications in Plants

Epigenetic regulatory mechanisms comprise DNA methylation, histone post-translational modifications and chromatin remodeling as pivotal components in plant developmental pathways. Multiple protein–protein, protein–DNA, protein–RNA and RNA–DNA complexes rule the deposition, reading and erasing of epigenetic marks on plant genomic DNA and chromatin. DNA methylation is crucial to maintain plant genome stability by avoiding the movement of widespread transposable elements and guiding particular gene expression at specific developmental stages or stress responses. Similarly, post-translational modifications at the N-terminus tails of core histones (H2A, H2B, H3, and H4) establish crosstalk with chromatin remodelers and transcription factors to control gene expression. Plant epigenetic regulation can also be mediated by small RNAs (sRNAs), which form a highly interactive network directing the silencing machinery to particular genomic regions.

### 3.1. Major Histone Modifications in Plants

Histone N-tails are prone to covalent modifications at different amino acid residues (predominantly lysine and arginine) by acetylation, methylation, monoubiquitination, phosphorylation, SUMOylation and ADP-ribosylation. Reversible histone acetylation by histone acetyltransferases (HATs) and deacetylation by histone deacetylases (HDACs), at particular lysine residues of H2A, H2B, H3 and H4, play a crucial role in gene activity regulation [24]. Histone hyperacetylation relaxes the chromatin structure and correlates with transcriptional activation, whereas hypoacetylation associates with compacted chromatin and gene repression. Such dynamics are essential to induce genome-wide chromatin modifications and specific gene expression changes for several plant biological processes [25]. The presence of H3K9Ac and H3K27Ac in regions near genes or gene bodies has been associated with active transcription in plants. During development, or in response to stress, acetylation marks are removed by HDACs to promote silencing. *Arabidopsis thaliana* HDACs are grouped in three families: RPD3/HDA1-like and Sirtuin 2-like, and HD2, and the role of some members has been studied in detail during seed development and germination [26]. Interestingly, HDACs could act in concert with methylation repressive mark deposition on the residue [27].

Histone lysine methylation (mono-, di- or tri-methylation) has been associated with both increase and decrease in gene expression. Histone lysine methyl transferases (HKMTases) display an evolutionarily conserved catalytic region, SET (**S**uppressor of variegation, **E**nhancer of zeste and **T**rithorax) and give rise to specific epigenetic marks [27]. ARABIDOPSIS HOMOLOG TRITHORAX 1-5 (ATX1-5), ARABIDOPSIS TRITHORAX-RELATED 1-7 (ATXR1-7), and ABSENT, SMALL, OR HOMEOTIC DISCS 1 HOMOLOG 1-7 (ASHH1-7) are SET-domain methyltransferases acting on the K4 residue of histone H3 (H3K4) in *Arabidopsis* [28]. Due to their action, mono-, di- or tri-methylated forms of H3K4 can be found in gene rich and promoter regions (active chromatin), but not in heterochromatic regions (transposable elements and repetitive DNA) [29]. Nevertheless, a recent report proposed that this modification could also work as a novel repressive mark [30].

Methylation of lysine 36 in histone H3 (H3K36) has been associated with active expression by transcription elongation promotion in plants [31]. The *Arabidopsis* SET DOMAIN GROUP 8 (SDG8) is a SET-domain containing the protein homolog to yeast SET-2 and is required for H3K36 di- and tri- methylation. Reduction of H3K36me2 and H3K36me3 in *sgd8 Arabidopsis* mutants causes pleiotropic effects, including flowering time delay, reduced plant size and fertility and deregulation of light/carbon responsive genes [32,33]. In addition, the deposition of H3K36me3 in a group of rice genes has been implicated in floral organ identity, regulation of pollen tube growth, and hormonal-mediated growth and development [34].

The H3K27 tri-methylation (H3K27me3) acts as major repressive mark for gene expression in *Arabidopsis* and other species to finely tune gene expression across different tissues and during development [35,36]. While H3K27me3 repressive patches are enriched at promoter regions and could span several inactive genes in animals, in plant genomes the mark spreads across the gene body, with higher accumulation at the transcription start site [37]. This creates an epigenetic landscape of gradually fading H3K27me3 islands over transcribed regions [38]. Slight, but significant, modifications on such distribution were detected under mild salt treatment and drought resulting in island shortening or fragmentation [39]. Interestingly, mark removal primarily occurred at island edges and in valleys within islands, thereby allowing the expression of certain genes to contend with future stressor events. H3K27me3 is deposited by PcG proteins, which form POLYCOMB REPRESSIVE COMPLEX (PRC) 1 and 2 [40]. The *Arabidopsis* genome contains functionally conserved homologues for the PRC2 core components but lacks some PRC1 core homologues that recognize the H3K27me3 mark [41]. Instead, LIKE HETEROCHROMATIN PROTEIN 1 (LHP1), homologous to the animal H3K9me2/H3K9me3-biding HETEROCHROMATIN PROTEIN 1 (HP1), acts as an H3K27me3-binding protein and promotes the recruitment of other PRC1-like components in *Arabidopsis* [36,42].

Plant H3K9me2 is significantly enriched at chromocenters and transposable elements (TEs) where the mark delimits heterochromatin [43]. In addition, H3K9me2 levels tightly correlate with regions that differ in DNA methylation [44]. The *Arabidopsis* genome contains multiple homologues of H3K9 methyl transferase that belong to the Su(var)3-9 family (HP1 included), SUV HOMOLOGS (SUVH1-5) and SUV-RELATED HOMOLOG (SUVR4-7) [27]. Histone marks are recognized by ATP-dependent chromatin remodelers that mediate nucleosome sliding, histone variant replacement or nucleosome reconstruction to regulate specific gene expression during plant growth and development [45].

### 3.2. DNA Methylation in Plants

Cytosine methylation is a conserved epigenetic mark for gene expression regulation, genome stability and gene imprinting in plants, fungi and animals. In plants, DNA methylation is found in CG, CHG and CHH sequence contexts (where H may be A, C or T) and is highly distributed over TEs, repetitive DNA, pericentromeric regions and in small patches between genes. This mark plays a pivotal role in silencing transcription of these regions [46]. However, DNA methylation can also trigger transcriptional gene silencing (TGS) when present at gene regulatory regions or affect mRNA processing, splicing and alternative polyadenylation when present within introns [47]. Additionally, CG methylation can be found over the bodies of housekeeping or constitutively expressed genes in many plant species without a precise functional role [48].

Dynamic regulation is required for the establishment, maintenance and removal of DNA methylation. Establishment involves an RNA-directed DNA methylation (RdDM) pathway that catalyzes de novo methylation in all sequence contexts through DOMAINS REARRANGED METHYLTRANSFERASE 2 (DRM2). Several pathways have been proposed for RdDM and their interplay is not completely understood. In *Arabidopsis*, canonical RdDM requires plant-specific RNA polymerases IV (POL IV) and V (POL V), RNA-DEPENDENT RNA POLYMERASE 2 (RDR2), DICER-LIKE 3 (DCL3) and ARGONAUTE (AGO4) proteins, as well as histone readers and chromatin remodelers (reviewed in [49]).

Transcripts produced by POL IV are converted to double-stranded RNA (dsRNA) by RDR2 and processed by DCL3 to originate 24-nt-long small interferent RNAs (siRNAs). These siRNAs are bound by AGO4 and recruited to regions transcribed by POL V, complementary in sequence to the siRNA-AGO4. The complex recruits DRM2 and triggers de novo DNA methylation [46,50]. The maintenance of this mark depends on its context. CG methylation is maintained by METHYLTRANSFERASE 1 (MET1), right after DNA replication. On the other hand, CHG and CHH methylation are maintained by CHROMOMETHYLASE 3 (CMT3) and 2 (CMT2), respectively [46]. Interestingly, CHG methylation is coordinated with H3K9me2 deposition and together they reinforce the repressive epigenetic status [51].

Alterations in DNA methyltransferases and RdDM pathways, or deficits in methyl group donors, lead to failure of DNA methylation level maintenance, also known as passive DNA demethylation. However, plants also use active demethylation by REPRESSOR OF SILENCING 1 (ROS1), DEMETER (DME) and DEMETER LIKE 2/3 (DML2/3), which directly remove methylated cytosines in any context by base excision repair and prevent hypermethylation at multiple regions [52]. Interplay between DNA methylation/demethylation and other epigenetic modifications are vital for various developmental plant processes, including the regulation of seed development, pollen tube formation, fruit ripening and stomatal development [46,52].

### 3.3. Small Non-Coding RNA Epigenetic Regulation

As anticipated in the previous section, the interaction between siRNAs, DNA methylation and histone modification guides the transcriptional silencing of DNA at TEs, repetitive regions or on specific genes. However, sRNA roles transit from post-transcriptional gene silencing (PTGS) to TGS and intersect through their biogenesis pathways (reviewed in [53]). sRNAs involved in TGS are also termed heterochromatic siRNAs (hcsiRNAs), while those acting in PTGS are represented by microRNAs (miRNAs), natural anti-sense siRNAs (natsiRNAs), phased siRNAs (phasiRNAs) and *trans*-acting siRNAs (tasiRNAs). miRNAs and tasiRNAs exert their function by inducing target cleavage and/or translational repression. Despite their mode of action, miRNAs and tasiRNAs target several central transcription factors and chromatin remodelers and are hence implicated as pivotal controllers of transcriptional and epigenetic regulators in diverse plant developmental processes.

Plant hc-siRNAs mapped to repeat-rich loci and TEs represent the most abundant sRNA class in many genomes. Their presence relates to the DNA methylome and serves as the “guardian” of plant genome architecture [54]. Additionally, hc-siRNAs and RdDM participate in spatio-temporal repression of genes [55], mobile epigenetic regulation [56] and epigenetic reprogramming during gametogenesis [57]. New insights suggest versatility in their mode of action. It was found that Pol IV switches to produce 21–22 nt hc-siRNAs in pollen and sporophytic tissues, where they are loaded to AGO1 instead to AGO4 to exert PTGS by cleavage of target RNAs transcribed from genomic regions [58]. However, further research is needed to shed light on their role in particular gene regulation.

## 4. Chromatin and Epigenetic Dynamics in Seed Development and Germination

### 4.1. Histone Modification Roles in Key Gene Expression Control

**Figure 2:**
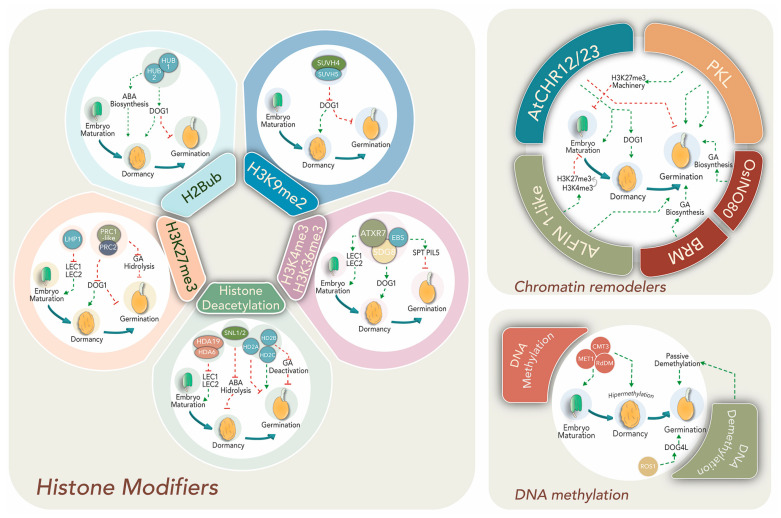
Principal epigenetic modifications and effectors regulate dormancy and germination.

Late embryogenesis, seed maturation and dormancy entrance are accompanied by chromatin compaction and decreased transcriptional activity [59]. Despite this, transcription of maturation- and dormancy-related genes increases, suggesting epigenetic mechanisms might enable particular gene expression within dense chromatin. Histone mark-mediated epigenetic regulation is required for dynamic gene expression changes to promote the switch from mature embryo to seedling developmental programs (Figure 2, left panel).

Crosstalk between hormones and transcriptional factors establishes a complex network regulation with epigenetic machineries such as histone modifiers (left panel), chromatin remodelers (right upper panel), and DNA methylation (right lower panel). Epigenetic effectors, regulatory pathways, specific genes and phytohormone relationships are discussed within the text.

Transcription factors LEAFY COTYLEDON 1 and 2 (LEC1 and 2), FUSCA 3 (FUS3) and ABSCISIC ACID INSENSITIVE 3 (ABI3) are pivotal regulators of seed maturation in various angiosperms including important crop species [60]. Their expression is promoted by the presence of H3K4me3 at promoter regions. Mutants affected in H3K4 and H3K36 methyltransferases, ATXR7 and SDG8, or the H3K4me2/3 “reader”, EARLY BOLTING IN SHORT DAY (EBS), present altered seed dormancy and germination [61,62]. Upon germination, the activation mark is substituted by LHP1-deposited H3K27me3 to silence their expression [63]. Moreover, acetylation of H3K9 and H3K4 at 5′ regions of *LEC1*, *LEC2* and *FUS3* genes is reduced by HDACs (HDA6 and HDA19) at postgerminative stages in *Arabidopsis* [26]. Interestingly, the expression of HDA6 and 19 is induced by ET and JA. In addition, HDA6 interacts with master transcription factors involved in both phytohormone signaling pathways and represses the transcription of their target genes by deacetylation [64].

Epigenetic regulators and histone modifiers act in concert with tight ABA/GA balance to direct dormancy or germination. Transcription factors SPATULA (SPT) and PHYTOCROME INTERACTING FACTOR3-LIKE5 (PIL5) suppress germination and GA biosynthetic genes in dormant seeds [65]. Therefore, their regulatory regions are enriched in H3K4me3, H3K36me3 and H3K9Ac in order to allow high expression before germination [66].

A well-documented master dormancy regulator is DELAY OF GERMINATION-1 (DOG1) that acts in concert with ABA-signaling during seed maturation. Loss of DOG1 function results in no dormancy and absence of endogenous ABA. *DOG1* accumulation is higher 14–16 days after pollination and disappears during after-ripening and imbibition [67,68]. Its expression is promoted in dormant seeds by the H3K4me3 activation mark, while the repressing mark H3K27me3 prevails in germinating seeds due to the action of PRC1-like and PRC2 complexes [63]. In addition, SUVH4 and SUVH5 repress *DOG1* via H3K9me2 deposition during light-mediated seed germination in *Arabidopsis* [69,70]. Seed dormancy is also altered by mutations of other histone modifiers, such as HISTONE MONOUBIQUITINATION1 and 2 (HUB1/HUB2), which modify H2B by monoubiquitination to increase transcription initiation and early elongation. *DOG1* and other dormancy-related genes display reduced transcript levels in *hub1* mutant seeds, suggesting that HUB1 may be acting upstream [71].

ATP-dependent chromatin remodelers cooperate to achieve the dormant stage (Figure 2; right upper panel). Over-expression of *Arabisopsis* SWI/SNF2 remodelers AtCHR12 and AtCHR23 reduce the frequency of seed germination and such reduction is intensified under stress conditions [72]. Moreover, decrease of germination is accompanied by increases in the RNA levels of *DOG1* and other seed maturation transcripts. This evidences a functional link between chromatin modifiers and regulatory networks operating towards seed maturation and germination. More importantly, these remodelers are also presumed to function as central transducers of environmental cues like temperature within the process [72].

Histone deacetylation exerts both positive and negative effects on dormancy, depending on the genes regulated by this mark (Figure 2). For example, a positive effect on dormancy was observed through deacetylation of ABA-hydrolytic and some ethylene-related genes. Seeds of mutants for SIN3-LIKE1 and 2 (SNL1/2) proteins, which physically interact with HDA19 to remove histone tail lysine acetylation, exhibit reduced dormancy similar to the *hda19* mutant [73]. Therefore, SNL-HDA19 promotes ABA increase and SNL expression declines together with ABA levels during germination [74,75]. Moreover, SNL1 and SNL2 suppress ET signaling through the deacetylation of H3K18 associated with ethylene-related genes. Moreover, the *snl1* and *snl2* knockouts show reduced dormancy and enhanced response phenotype to ET [73]. Conversely, other deacetylase activity might reduce dormancy through repressing the expression of GA deactivation genes like GA2ox2 or upstream negative regulators of GA biosynthetic genes like GA3ox1 and GA3ox2 [74,76]. Supporting this, *Arabidopsis* dormant accessions exhibit low *HISTONE DEACETYLASE 2B* (*HD2B*) expression, but when transformed with the HD2B gene from a less-dormant accession, displayed a reduction in mature seed dormancy [76].

Germination requires histone acetylation for activation of genes associated with seedling growth and histone deacetylation for silencing embryonic traits (Figure 2). Overexpression of the deacetylation complex HDC1 improves germination under ABA and paclobutrazol (a GA synthesis inhibitor) treatments [77]. Besides, deacetylases HD2A and HD2C have been proposed to exert contrary effects on the germination rate. HD2A downregulates germination in response to glucose via a HEXOKINASE-1-independent pathway, but HD2C supports germination through ABA gene response regulation [78]. This could explain the simultaneous increase of both HATs and HDACs during germination in order to establish multiple interactions and execute diverse functions during the seed performance [79,80]. 

Changes in the transcriptional landscape from dormancy to germination begin with seed imbibition. The highly condensed chromatin achieved during seed maturation becomes relaxed, with progressive increases in RNA synthesis and DNA repair [59,81]. The ATP-dependent chromatin remodeler PICKLE (PKL) is involved in epigenetic control of gene expression during *Arabidopsis* seed germination (Figure 2) and at several post-germination stages [82]. Recently, it was shown that PKL acts in concert with the SWR1-family remodeler PHOTOPERIOD INDEPENDENT EARLY FLOWERING1 (PIE1), which incorporates the histone variant H2A.Z to provide H3K27me3 homeostasis during seedling growth [83]. Moreover, PKL represses the expression of *ABI3* and *ABI5*, positive regulators of the ABA pathway, hence reducing ABA signaling and promoting germination [84]. In *pkl* plants, master seed maturation regulators *LEC1*, *LEC2* and *FUS3* display higher transcript levels [59]. 

Suppression of the embryo maturation gene expression during germination also depends on the repressive PRC2 complex. Mutants of *FERTILIZATION INDEPENDENT ENDOSPERM* (*FIE*), an essential component of PRC2, display abnormal seed development, increased dormancy, germination defects and altered vernalization [85,86]. Genomic ChIP-seq characterization of these mutants showed reduction of the H3K27me3 mark on many genes, including positive ABA regulators and negative GA regulators, evidencing its role in dormancy suppression and germination progression [85]. A chromatin state switch by H3K4me3-to-H3K27me3 replacement in seed-developmental genes like *ABI3*, *DOG1* and *CRUCIFERIN 3 (CRU3)* is promoted by the interaction between PHD-domain H3K4me3-binding ALFIN1-like proteins (ALs) and core PRC1-like components and LHP1 to modify gene expression and stimulate germination [63].

The positive epigenetic regulation in germination depends on ATP-dependent-chromatin-remodelers like OsINO80 and BRAHMA (BRM), which stimulate GA biosynthesis and seed germination (Figure 2, upper right panel) [52,87]. In rice, OsINO80 directly binds to chromatin at the GA biosynthesis genes *CPS1* and *GA3ox2* and promotes nucleosome remodeling with histone variant H2A.Z to enhance their expression and increase the GA level. *Osino80*-knockdown mutants display retarded seed germination, dwarfism, late flowering and impaired reproductive development. Interestingly, BRM also modulates the nucleosome occupancy and histone mark deposition in regulatory regions of genes involved in dormancy to germination switches like ABA response (*PPC2*) [88] and *MIR156* [89].

### 4.2. DNA Methylation Reprogramming During Germination

DNA methylation represents a major epigenetic regulation for gene expression reprogramming during seed development, dormancy and germination. While many studies have described the dynamic DNA methylation/demethylation process during plant reproductive growth (comprehensively reviewed in [90,91]), a few recent reports have approached what happens upon seed imbibition [92,93,94]. After fertilization, the MET1, CMT3 and RdDM pathways are highly active, leading to global hypermethylation at embryo maturation and dormancy in *Arabidopsis* (Figure 2, lower right panel). However, the methylation level, particularly in a CHH context, is lower in the endosperm than in the embryo [95]. It has been proposed that hypomethylation of some TEs in the endosperm accounts for sRNA expression from these sequences acting as mobile signals to the embryo, where they reinforce silencing at homologous sequences for the future plant.

The methylation status of particular genome regions and its correlation with gene expression may differ between plant species. For example, all methylation contexts are frequently present in protein-coding gene bodies in maize [96], whereas rice presents almost exclusively CG methylation at these regions [57]. Maize CG methylation within gene coding regions correlated with active transcription elongation, but CHG and CHH methylations coincided with lower transcription rates at the same sequences.

Throughout dormancy acquisition, the dry seed methylome exhibits one of the higher mCHH levels compared to other tissues and cells. Interestingly, this mark accumulates within TE-rich regions and influences the expression of nearby genes. Changes in the methylation status during *Arabidopsis* germination, especially occur for genes required for transcription regulation, RNA processing and protein modifications [93]. Therefore, the extensive gain of RdDM- and CMT2-dependent CHH methylation within TEs during seed development and desiccation is lost during germination [97]. Global hypomethylation is not impaired in demethylase mutants, indicating that passive demethylation probably conducts the reduction from dry seed to post-germinative stages.

Predominant demethylation in germinating seeds was also observed in other species like *Triticum aestivum* [98], *Capsicum annum* L [99] and *Castanea sativa* [100]. Interestingly, a high plasticity in DNA methylation in the CHH context was observed for rice germinating seeds in response to oxygen availability [101]. Rice seeds germinated similarly under both aerobic and anaerobic conditions and displayed hypomethylated regions with respect to dry seeds. Surprisingly, some of these regions became hypermethylated at CHH within 24 h upon oxygen supply for the anaerobic seedlings. 

Hypermethylation during seed development and hypomethylation during germination are partially associated with germination-related gene expression [97]. Concurrently, local demethylation can regulate both seed dormancy and germination genetic pathways. For example, *DOG4L*, a paralogous gene of *DOG1*, promotes germination and negatively controls dormancy and ABA sensitivity. Interestingly, this gene exhibits differential promoter methylation between alleles, being expressed only from the maternal allele [102]. However, demethylase ROS1 prevents excessive methylation at the promotor of the paternal allele and allows the expression of *DOG4L* (Figure 2). Both *ros1* and *dog4l* mutants exhibit enhanced dormancy, which is released after *DOG4L* ectopic overexpression [102].

Dynamic DNA methylation also occurs under different treatments that break the dormant state. In almonds (*Prunus dulcis*), exposing seeds to low temperatures contrives to dormancy release, depending on genotype. Seeds with promptly released dormancy upon cold stratification presented hypermethylation at regulatory regions of AUXIN RESPONSE FACTORS (ARFs) and LATE EMBRYOGENESIS ACCUMULATED (LEA) encoding genes, suggesting that dry seed regulatory network components are suppressed by DNA methylation in preparation for germination [103].

All this evidence reflects a crucial role of DNA methylation in controlling the developmental switches during seed growth and germination. Potential agronomic applications could involve the management of appropriate storage conditions or seed pretreatments that impact DNA methylation towards dormancy or germination. Pre-harvesting sprouting prevention or the enhancement of synchronized germination for higher yields are potential future areas to explore for biotechnological purposes.

### 4.3. Small RNA Roles in Germination: From Memory to New Perceptions

Small RNAs are central effectors of gene expression during the life cycle of plants. From embryo development to vegetative identity acquisition, sRNAs regulate their targets and support developmental phase transitions. Particularly, the miRNA group has essential roles in embryogenesis, germination, organ patterning, flowering, hormone signaling and stress perception (reviewed in [104,105,106]). Most miRNAs target transcription factor networks and establish gradients of gene expression to define particular cell fate above thresholds. In addition, they often connect to other sRNA pathways as is the case for miR390-tasiRNAs-ARF3/4 [107]. Still, their regulatory role in seed germination has not been deeply explored and from the many miRNAs showing expression changes upon seed imbibition, only a few have been related to particular function at molecular and physiological levels (Table 1) [106].

Mutations in *DCL1*, required for miRNA biogenesis, affect the very early embryogenesis program. Null mutant embryos fail to develop beyond the globular stage and observed morphological defects are due to poor accumulation of embryonic miRNAs and upregulation of their targets, which should become repressed for proper differentiation [119,120]. Altered expression patterns lead to developmental irregularities like premature cell differentiation, changes in cell fate, modification of early organization and meristem alterations [119,121]. Similar effects were observed for mutations in other genes of the miRNA biogenesis pathway [122].

Several important regulators of seed maturation have been proposed to be indirectly controlled by specific miRNAs. In *dcl1* mutant embryos, *LEC2* and *FUS3* appear as upregulated transcripts, whereas repressors such as histone deacetylases were downregulated [122]. Moreover, it was found that at the early globular stage miR156 should repress the *SQUAMOSA BINDING PROTEIN-LIKE* (*SPL*) transcription factor family, which might inhibit the expression of repressor of maturation genes to control the proper induction of seed maturation and dormant stage timing [108,119].

miRNAs have been related to both activators and repressors of seed dormancy and germination in multiple species [106,112,120,123]. A particularly interesting regulation is exerted by the dormancy regulator DOG1 on miR156 and miR172 balance [109]. The interplay between these two miRNAs is known to support vegetative to reproductive transition in *Arabidopsis* [124]. miR156 is highly expressed during the plant juvenile stage and its decrease determines miR172 expression to promote the reproductive stage. Huo et al. [109] found that miR156 relates to dormancy establishment and depends on DOG1 for its correct processing from precursor. On the other hand, reduction of miR156 results in *SPL* increased levels, promoting miR172 expression and germination. Therefore, the balance between miR156 and miR172 levels sets the dormancy length and germination promotion in response to environmental conditions [109].

Another miRNA related to dormancy/germination is miR159. It targets *GAMYB*-like transcripts during seed maturation and acts in response to ABA and GA, depending on the tissue [110]. Impairment of miR159-mediated regulation on *AtMYB33* and *AtMYB101* increases germination sensitivity to ABA, suggesting it may act as negative regulator of ABA response during this process. Furthermore, downregulation of *GAMYB* by miR159 and GA promotes the aleurone-programmed cell death process required for germination [111].

Crosstalk between ABA and GA pathways and with other phytohormone signaling through dormancy to germination transition often involves miRNAs and other sRNAs acting as connecting bridges. For example, the AUX pathway involves the action of ARFs as transcriptional activators or repressors on specific gene promoters to elicit a physiological response. ARFs abundance is finely regulated by many mechanisms including miRNAs. Particularly, *ARF10* transcript levels are regulated by miR160 during germination and post-embryonic development [112]. When an miR160-cleavage-resistant form of *ARF10* is expressed and accumulates in *Arabidopsis*, the expression of several ABA response genes is enhanced, indicating that AUX may influence the ABA response pathway in *Arabidopsis*. It has been proposed that downregulation of *ARF10* by miR160 is required to reduce ABA sensitivity and allow radicle elongation during germination [113]. According to this, miR160-*ARF10* represents a crucial regulatory node that connects different hormone signaling pathways.

Other *ARFs* are regulated by miR390 during seed germination. miR390 connects two major classes of sRNAs, miRNAs and tasiRNAs, through promotion of *TAS3* transcript processing into functional tasiR-ARFs that target family members *ARF2*, *ARF3*, and *ARF4* [125]. When comparing miRNAs differentially accumulated between dry and imbibed Arabidopsis seeds, miR390b showed increased levels at 12 and 24 h after imbibition at both room temperature and 4 °C. Additionally, the transcript levels of *ARF2/3/4* were low at the early stages of germination and became high only after miR390 accumulation dropped at 48 h post-imbibition [114]. This proposes a potential role for the miR390-tasiR-ARF module in seed germination by adjusting the levels of some ARF transcription factors and their downward targets.

Links between hormonal pathways and sRNAs in germination have also been reported for other species. In wheat, a 22-nt miR9678 is specifically expressed in the scutellum of developing and germinating seeds, suggesting it has a potential role in regulating germination [115]. miR9678 targets a long non-coding *WHEAT SEED GERMINATION ASSOCIATED RNA* (*WSGAR*) and has classical characteristics of a 22-nt miRNA triggering biogenesis of phasiRNAs. Both miR9678 and miR9678-triggered phasiRNAs exhibit negative effects on germination and improvement of pre-harvesting sprouting. Interestingly, delay of germination seems to be promoted by direct regulation of miR9678 on targets involved in the GA biosynthetic pathway, rather than by transcript cleavage mediated by *WSGAR*-derived phasiRNAs, which possibly act through other unknown mechanisms [115]. In the same report it was demonstrated that miR9678 transcription depends on components of the ABA signaling pathway, suggesting miRNA-mediated crosstalk between ABA and GA in wheat seed germination.

While miRNAs mostly exert PTGS and do not promote direct epigenetic modifications, they could indirectly affect the epigenetic landscape by controlling the levels of transcript coding for specific chromatin remodelers or epigenetic modifiers. One example is represented by miR402, a relatively novel miRNA found to positively control seed germination, particularly under stress conditions [116]. miR402 targets demethylase *DML3* transcript and *dml3* mutant seeds exhibit accelerated germination under the same stress conditions. It was hypothesized that induction of miR402 by stress guides cleavage of *DML3*, whose reduction would help to maintain DNA methylation on genes with a negative role in seed germination [116]. However, further experiments are needed to confirm specific methylation changes due to miR402 overexpression.

In addition to miRNAs and tasiRNAs, other sRNAs could rule in dormancy and germination. In *Larix leptolepis*, the regulation of dormancy maintenance and release is accompanied by changes in siRNA populations. Dormant embryos exhibited higher accumulation of 24-nt siRNAs, while germinated embryos showed a bias toward 21-nt-long siRNAs. Apparently, this could be due to distinct expression levels of RDR2 and/or RDR6, indicating the contribution of different sRNA pathways to dormancy release [126]. On the other hand, many POL IV-transcribed siRNAs originated from maternal tissue during seed development and maturation have been found to regulate gene expression of endosperm genes [95,127]. These siRNAs represent up to 90% of the population during seed development and maturation [128]. Imprinting of *ALLANTOINASE* (*ALN*), an endosperm maternally expressed gene and negative regulator of dormancy, is controlled by methylation through 24-nt siRNAs and a non-canonical RdDM pathway to promote dormancy under cold [117]. Moreover, barley siRNAs guide the response to terminal drought stress during seed filling by targeting *CYTOKININ-OXIDASE2.1* (*HvCKX2.1*), a cytokinin oxidase/dehydrogenase (CKX; EC.1.5.99.12) involved in the degradation of cytokinin and control of germination timing [118]. When terminal drought is applied to immature seeds, stress specific 24-nt siRNA production increases DNA methylation at the *HvCKX2.1* promoter region, decreasing its mRNA levels and further affecting germination rate and shoot emergence, probably by accumulation of cytokinin ribosides.

## 5. Conclusions and Perspectives

In this work, we have presented an overview of the epigenetic regulators and sRNAs involved in central processes during seed maturation, dormancy and germination in several plant species. Major transitions during the life cycle of plants require fine-tuning regulation at the molecular and cellular levels. To achieve this, the epigenetic landscape must reprogram. Our recent knowledge on dormancy to germination transition has been obtained mostly by genetic approaches in the model plant *Arabidopsis thaliana*. Lines exhibiting reduced or enhanced expression of specific genes involved in epigenetic signaling show altered dormant and germination phenotypes. However, epigenetic regulation is far more complex. The characterization of regulatory networks established between various chromatin modifiers with other epigenetic effectors and regulators (such as miRNAs and other sRNAs) has just started. Interestingly, epigenetic control is simultaneously exerted on dormancy and germination regulators, as well as on their own components, implying a feedback control of their activity. Undoubtedly, future works will focus on more than direct chromatin modification studies in order to identify those additional regulators and regulatory network nodes. Expanding the knowledge to agronomically relevant species such as maize, rice, wheat and others would not only contribute to the understanding of conservation and diversity in the epigenetic regulation of seed programs, but also to their impact on agronomical applications.

## Figures and Tables

**Table 1:** sRNAs involved in seed dormancy and germination.

sRNA	mRNA Target(s)	Dormancy ^a^	Germination ^a^	Description ^b^	References
miR156	*SPL13*	Positive		Proper induction of seed maturation and dormant stage timing.	[108]
miR172	*AP2* and *AP2*-like (*SZN*)		Positive	Seed development, seedling growth. SAM maintenance at post-seedling stages.	[74,109]
miR159	*AtMYB33*; *AtMYB101*		Positive	Seed maturation. Negative regulation of ABA response. Aleurone programmed cell death.	[110,111]
miR160	*ARF10*		Positive	ABA-Auxin crosstalk. ABA sensitivity decreases in mature seeds. Switch to germination mode. Radicle elongation.	[112,113]
miR390 tasiR-ARFs	*ARF2*, *ARF3*, *ARF4*	-	-	Seed maturation and endosperm development. Lateral root formation.	[114]
miR9678	*WSGAR*		Negative	Delay of germination. Inhibition of GA biosynthetic pathway.	[115]
miR402	*DML3*		Negative	Increased germination rate under stress conditions (salinity and cold stress).	[116]
24-nt siRNAs	*ALN*	Positive		Seed development, dormancy regulation under cold stress.	[117]
24-nt siRNAs	*HvCKX2.1*		Negative	Terminal drought stress seed filling. Germination and shoot emergence delay.	[118]

^a^ Demonstrated stimulatory or inhibitory effect on dormancy or germination. ^b^ Description of particular regulation on seed developmental or physiological processes.

## Data Availability

Not applicable.
